# Federated learning for cognitive impairment detection using speech data

**DOI:** 10.3389/frai.2025.1662859

**Published:** 2025-10-09

**Authors:** Josep Blazquez-Folch, María Limones Andrade, Berta Calm, Juan Miguel Auñón García, Montserrat Alegret, Nathalia Muñoz, Amanda Cano, Victoria Fernández, Fernando García-Gutiérrez, Itziar De Rojas, Pablo García-González, Clàudia Olivé, Raquel Puerta, María Capdevila-Bayo, Álvaro Muñoz-Morales, Paula Bayón-Buján, Andrea Miguel, Laura Montrreal, Ana Espinosa, Pilar Sanz-Cartagena, Maitee Rosende-Roca, Carla Zaldua, Peru Gabirondo, Yahveth Cantero-Fortiz, Miren Jone Gurruchaga, Lluis Tarraga, Mercè Boada, Agustín Ruiz, Marta Marquié, Sergi Valero

**Affiliations:** ^1^Ace Alzheimer Center Barcelona - Universitat Internacional de Catalunya, Barcelona, Spain; ^2^Department of Artificial Intelligence and Big Data, GMV, Madrid, Spain; ^3^Networking Research Center on Neurodegenerative Diseases (CIBERNED), Instituto de Salud Carlos III, Madrid, Spain; ^4^Accexible Impacto s.l., Urduliz, Bizkaia, Spain; ^5^Glenn Biggs Institute for Alzheimer’s & Neurodegenerative Diseases, University of Texas Health Science Center, San Antonio, TX, United States; ^6^Department of Microbiology, Immunology and Molecular Genetics, Long School of Medicine. University of Texas Health Science Center, San Antonio, TX, United States

**Keywords:** deep learning, Alzheimer’s disease, cognitive impairments, speech acoustics, federated learning

## Abstract

**Introduction:**

In Alzheimer’s disease (AD) research, clinical, neuroimaging, genetic, and biomarker data are vital for advancing its understanding and treatment. However, privacy concerns and limited datasets complicate data sharing. Federated learning (FL) offers a solution by enabling collaborative research while preserving data privacy.

**Methods:**

This study analyzed data from patients assessed at the Memory Unit of the Ace Alzheimer Center Barcelona who completed a standardized digital speech protocol. Acoustic features extracted from these recordings were used to distinguish between cognitively unimpaired (CU) and cognitively impaired (CI) individuals. The aim was to evaluate how data heterogeneity impacted the FL model performance across three scenarios: (1) equal contributions and class ratios, (2) unequal contributions, and (3) imbalanced class ratios. In each scenario, the performance of local models trained using an MLP feed-forward neural network on institutional data was analyzed and compared to a global model created by aggregating these local models using Federated Averaging (FedAvg) and Iterative Data Aggregation (IDA).

**Results:**

The cohort included 2,239 participants: 221 CU individuals (mean age 66.8, 64.7% female) and 2,018 CI subjects, comprising 1,219 with mild cognitive impairment (mean age 74.3, 61.9% female) and 799 with mild AD dementia (mean age 80.8, 64.8% female). In scenarios 1 and 3, FL provided modest gains in accuracy and AUC. In scenario 2, FL markedly improved performance for the smaller dataset (balanced accuracy rising from 0.51 to 0.80) while preserving 0.86 accuracy in the larger dataset, highlighting scalability across heterogeneous conditions.

**Conclusion:**

These findings demonstrate the potential of FL to enable collaborative modeling of speech-based biomarkers for cognitive impairment detection, even under conditions of data imbalance and institutional disparity. This work highlights FL as a scalable and privacy-preserving approach for advancing digital health research in neurodegenerative diseases.

## 1 Introduction

Dementia, particularly Alzheimer’s disease (AD), poses a growing global health challenge among the aging population. In the United States alone, over 6.9 million individuals aged 65 and older are estimated to be living with AD, a number projected to nearly double to 13.8 million by 2060 (Alzheimer's Association, 2024). Notably, 10.9% of this demographic is affected by the disease (Alzheimer's Association, 2024), underscoring its increasing impact.

Accurate diagnosis and effective treatment are complicated by the multifaceted nature of dementia, which is influenced by demographic, environmental, genetic, and biological factors. Artificial intelligence has emerged as a promising tool for cognitive-impairment screening, with machine learning (ML) and natural language processing applied to neuroimaging, electronic health records, speech, and other digital biomarkers. These methods show strong predictive performance and potential to improve diagnostic accuracy and efficiency, though concerns remain about misdiagnosis, confidentiality, and the psychological burden of screening (Wurtz et al., 2025; Arya et al., 2023). Among them, automated speech analysis has emerged as a non-invasive tool for detecting early cognitive decline (Hajjar et al., 2023). Language impairments associated with dementia manifest as difficulties in both speech production and comprehension (Bucks et al., 2000). However, large-scale studies leveraging speech data face significant obstacles, including concerns about patient privacy, speech de-identification, data sharing, and the need for collaboration across multiple research centers.

Federated learning (FL) has emerged as a promising ML approach for addressing these challenges by enabling multi-institutional data analysis while preserving patient confidentiality. Unlike traditional centralized methods, FL allows decentralized model training, ensuring sensitive information remains local (Kairouz et al., 2021; Hardy et al., 2017). This framework is particularly well-suited for privacy-sensitive domains such as healthcare, facilitating collaborative research while mitigating data security risks.

Although FL has shown promise in various healthcare applications (Teo et al., 2024), its use for speech-based dementia detection remains scarce and underdeveloped. Traditional centralized approaches face limitations due to restricted data access, ethical concerns, and biased representation, while variability in language, demographics, and recording conditions undermines generalization (García Gutiérrez et al., 2024; Sharafeldeen et al., 2025). FL offers a potential solution to these challenges, yet it introduces its own complexities. A key issue is data heterogeneity, where variations in dataset size and class distribution across institutions can negatively impact model fairness and overall performance (Hardy et al., 2017).

To our knowledge, until now there exists only a single prior work examining speech-based AD diagnosis within FL frameworks, which demonstrates its feasibility for AD detection from speech, with decentralized models achieving competitive performance while preserving privacy. However, these efforts rely on small, homogeneous datasets, and the impact of real-world heterogeneity on accuracy and fairness remains largely unexamined (Meerza et al., 2022).

To address this limitation, our study builds upon and significantly extends previous research by conducting a comprehensive evaluation of FL for dementia detection. Leveraging a substantially larger and more diverse dataset, we systematically evaluate model performance under various realistic scenarios, particularly emphasizing the challenges posed by data heterogeneity. Specifically, we investigate how data heterogeneity influences predictive accuracy and robustness. Our approach employs acoustic speech features within a multi-layer perceptron (MLP) neural network framework to distinguish between cognitively unimpaired (CU) and cognitively impaired (CI) individuals.

By examining the interplay between data variability and FL efficacy on a broader scale, this research provides deeper insights into optimizing FL models for real-world applications, ensuring both robust performance and equitable outcomes across diverse datasets.

## 2 Methods

### 2.1 Study participants

This study included data from individuals evaluated at the Memory Clinic from Ace Alzheimer Center Barcelona (Ace) between March 2022 and April 2023 (Table 1). All participants were diagnosed by a multidisciplinary team comprising neurologists, neuropsychologists, and social workers after completing a series of neurological, neuropsychological, and social assessments. Further details of the evaluation protocols are available elsewhere (García Gutiérrez et al., 2024; Alegret et al., 2011; Boada et al., 2014).

**Table 1:** Clinical and sociodemographic characteristics of the sample stratified by clinical condition.

Variable	CU	CI (MCI)	CI (ADD)
Sample size (%)	221 (9.9)	1,219 (54.4)	799 (35.7)
Age (mean, SD)	66.8 (10.2)	74.3 (9.4)	80.8 (6.8)
Sex (% Female)	64.7	61.9	64.8
Years of formal education (mean, SD)	12.3 (3.9)	8.8 (4.6)	7.7 (4.6)
MMSE (mean, SD)	29.2 (0.9)	26.9 (2.6)	21.6 (3.5)

CU, cognitively unimpaired; CI, cognitively impaired; MCI, mild cognitive impairment; ADD, Alzheimer’s disease dementia; MMSE, Mini–Mental State Examination; SD, standard deviation.

### 2.2 Speech protocol and automated speech analysis

All participants completed a brief speech protocol using the acceXible app platform on a tablet in a quiet and controlled environment under the supervision of a neuropsychologist. The protocol comprised two tasks: first, the description of The Cookie Theft Picture in approximately 1 min, a common language assessment test (Cummings, 2019); and second, a semantic verbal fluency test where participants listed as many animals as possible within 1 min.

Speech recordings were standardized to 16 kHz, with silence segments removed and noise reduction applied using the model in (Defossez et al., 2020). From the processed audio, in image description and verbal fluency tasks independently, various physical acoustic features were extracted, covering parameters related to frequency (pitch, jitter, formant 1,2, and 3 frequency and formant 1 bandwidth), signal energy/amplitude (shimmer, loudness and harmonics-to-noise ratio), and spectral parameters (alpha ratio, hammarberg index, spectral slope 0-500 Hz and 500-1500 Hz, formant 1,2, and 3 relative energy and harmonic difference H1-H2 and H1-A3). The extracted variables corresponded to those described in the extended Geneva Minimalistic Acoustic Parameter Set (eGeMAPS) (Eyben et al., 2015), a standardized set of acoustic parameters linked to physiological voice changes, often used in neurological disease contexts (García Gutiérrez et al., 2024; García Gutiérrez et al., 2023). Feature extraction was performed using the OpenSmile (v2.5.0) (Eyben et al., 2010) library, with a three-frame symmetric moving average across eighteen low-level descriptors, resulting in a total of 176 variables per participant. The full set of extracted features was carried forward to the analyses, with no dimensionality reduction or cross-site adjustments applied at the extraction stage. Further details on feature calculation are available in (Abyane et al., 2022).

### 2.3 Virtual scenarios

Using the initial data specified in Sections 2.1 and 2.2 from the Ace Alzheimer Center Barcelona, we simulated a FL environment involving two virtual independent institutions (Institution 1 and Institution 2). The institutions provided speech acoustic features, along with CU and CI labels, for their respective patients. Each institution uploaded its data to its computing node, and three scenarios were designed to comprehensively analyze how dataset characteristics affect model performance. These scenarios, detailed in Table 2, varied by dataset size and class proportions at each institution, enabling an evaluation of their impact on FL model effectiveness.

**Table 2:** Distribution of total, cognitive impaired (CI) and cognitive unimpaired (CU) cases per institution across different scenarios.

Scenario	Node	Percentage of total cases	Percentage of CI cases	Percentage of CU cases
Scenario 1	Node 1	50%	90%	10%
	Node 2	50%	90%	10%
Scenario 2	Node 1	10%	90%	10%
	Node 2	90%	90%	10%
Scenario 3	Node 1	67%	97%	3%
	Node 2	37%	78%	22%

Two different conditions were examined for each scenario: (1) individual training, where each institution trained its model using only local data, and (2) federated training, where both institutions collaborated to train a global model using FL.

In the individual training condition, each institution trained a model exclusively on its local data and evaluated on both its own and the other institution’s data. This setup, yielding two distinct models trained on separate datasets, allowed for the assessment of how data volume and quality variations at each institution influenced model performance, particularly in scenarios of limited data availability. Conversely, in the federated training condition, a collaborative model was trained on data from both institutions, leveraging data diversity to enhance model robustness and generalizability. This approach capitalized on the combined dataset, improving the model’s overall performance.

#### 2.3.1 Scenario 1: uniform sample size

In scenario 1, each institution contributes an equal amount of data with a uniform ratio of CI to CU cases. This setup ensures consistency in sample size and class distribution across institutions, allowing for an evaluation of the FL model’s performance under balanced and evenly distributed data conditions.

#### 2.3.2 Scenario 2: varying sample size

In scenario 2, institutions have varying data sizes, but the ratio of CI to CU cases remains consistent. This setup evaluates the FL model’s performance under imbalanced data distribution, focusing on its robustness and generalizability across institutions with unequal dataset sizes.

#### 2.3.3 Scenario 3: imbalanced class ratio

In scenario 3, institutions differ in their class distributions, with one having more CI cases and another more CU cases. This setup examines the FL model’s ability to handle class distribution imbalances, reflecting real-world challenges where institutional data often lacks uniformity.

### 2.4 Neural network architecture and federated learning setup

In FL, designing an effective network requires balancing local device limitations, like computational capacity and data availability, with the need for coordinated global model updates to ensure convergence (Abyane et al., 2022). The goal is not necessarily the highest model accuracy, but overcoming challenges of cross-institution collaboration while maintaining data privacy. This highlights the advantages and challenges of secure, collaborative data sharing in heterogeneous environments, along with issues of privacy and model convergence.

**Figure 1:**
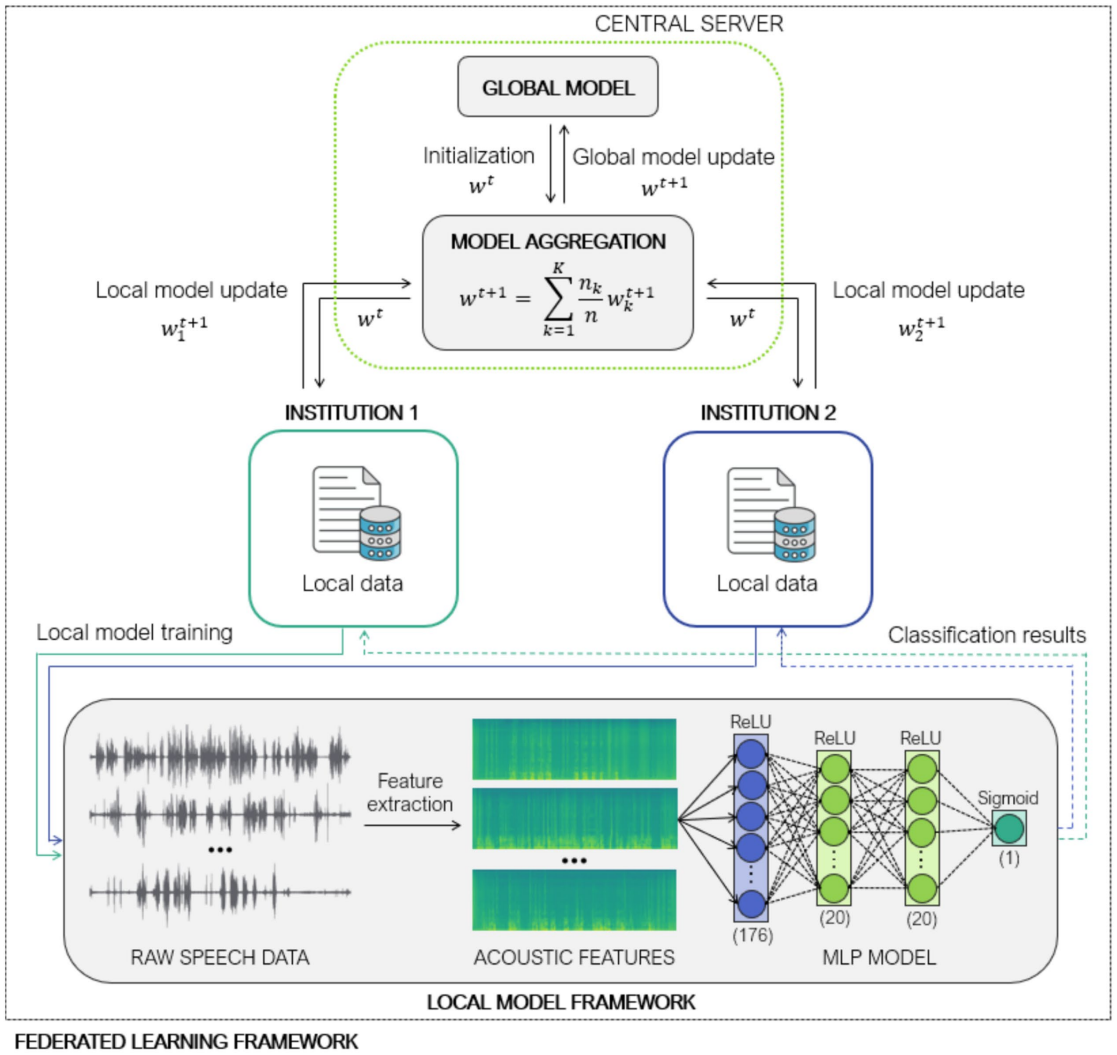
Federated learning architecture using Federated Averaging (FedAvg) and Iterative Data Aggregation (IDA) across two institutions for decentralized multi-layer perceptron-based classification of cognitive impairment from speech-derived acoustic features.

To address these challenges, we employed a feed-forward MLP as the local model architecture. The MLP consisted of an input layer with 176 neurons, each corresponding to an input acoustic feature, followed by two hidden layers with 20 neurons each, utilizing ReLU activation functions. The output layer comprised a single neuron with a sigmoid activation function for binary classification. The network architecture is illustrated in Figure 1 in the local model framework.

Local models were trained using stochastic gradient descent (Wojtowytsch, 2021) with an initial learning rate of 0.01. To enhance convergence stability, an exponential learning rate scheduler with a decay factor of 0.95 per step was applied. Training was conducted with a batch size of 32 over 150 epochs. Hyperparameters were not systematically tuned but chosen based on initial configurations with manual adjustments. The binary cross-entropy loss function was employed, appropriate for binary classification tasks. The dataset was split into training (70%) and test (30%) sets with no overlap (training ∩ test = ∅). Performance was evaluated using standard classification metrics, including the Area Under the ROC Curve (AUC-ROC).

Rather than employing explicit regularization techniques such as Dropout, Batch Normalization, or weight decay, overfitting control was achieved through the compact architecture of the network, monitoring of validation performance, and the use of class-balancing weights in the loss function. To account for class imbalance, weights were initialized inversely proportional to class frequency, normalized by a factor of 2, thereby assigning greater importance to the minority class. These weights were dynamically applied at each batch to maintain balanced contributions throughout training.

To aggregate locally trained models, we employed the Federated Averaging (FedAvg) algorithm (McMahan et al., 2017), which computes a weighted average of local model parameters, assigning weights proportional to the number of training samples per client. Mathematically, this aggregation is defined as:


$$w^{t+1}=\sum_{k=1}^{K}\frac{n_{k}}{n}w_{k}^{t+1}$$

where $w^{t+1}$ denotes the updated global model parameters, $w_{k}^{t+1}$ represents the parameters obtained from client k after local training, nk is the number of training samples held by client k , $n=\sum_{k=1}^{K}n_{k}$ is the total number of training samples, and K is the number of participating clients (with K=2 in our study). This approach assumes that clients with larger datasets generate more reliable model updates, and thus should exert greater influence on the aggregated global model.

However, FedAvg is known to be vulnerable in federated settings characterized by non-independent and identically distributed (non-IID) data or unbalanced sample sizes, where local models can diverge significantly due to class imbalances or other localized biases. As a result, weighting updates solely based on data volume can inadvertently amplify the influence of low-quality or even adversarial model updates, ultimately degrading the robustness and generalization ability of the global model.

To mitigate this issue, we incorporated the Iterative Data Aggregation (IDA) algorithm (Yeganeh et al., 2020), a robust aggregation strategy that assigns weights based on the similarity of local models to the average model. Specifically, IDA downweights local models that deviate significantly from the average by computing weights inversely proportional to their ℓ1-distance from the average model. The weighting coefficient for each local model k is given by:


$$\alpha_{k}=\frac{1}{Z}{\left(w_{\mathrm{Avg}}^{t}-{w_{k}^{t}}_{1}+\varepsilon \right)}^{-1}$$

where $w_{\mathrm{Avg}}^{t}=\frac{1}{K}\sum_{k=1}^{K}w_{k}^{t}$ is the average of the local models at round t , Z is a normalization factor to ensure $\alpha_{k}$ all sum 1 and ε is a small constant to prevent division by zero. The aggregation process is then defined as:


$$w^{t+1}=\sum_{k=1}^{K}\alpha_{k}w_{k}^{t+1}$$

By relying on model similarity rather than sample count, IDA enhances robustness to statistical heterogeneity, noisy updates, and outlier models, which are common in real-world federated scenarios. The entire aggregation process is illustrated in Figure 1.

#### 2.4.1 Development

The FL system was implemented using Python 3.11.9 with uTile (GMV, 2024), which internally leverages PyTorch 2.0.0 for deep learning model development. In this setup, uTile coordinates training across multiple decentralized nodes, allowing each to train locally on its data while sharing model updates to a central aggregator. This approach enhances data privacy by avoiding the transfer of raw data between nodes and the central server. Instead of transmitting raw data, only model weights or gradients are communicated, preserving data privacy while enabling collaborative learning.

## 3 Results

Data from 2,239 participants were analyzed: 221 individuals who were CU, showing no objective cognitive or functional impairment (CDR = 0) (Jessen et al., 2014) and 2,018 participants who exhibited CI, including patients with mild cognitive impairment (MCI) (*n* = 1,219, CDR = 0.5) (Petersen, 2004) and dementia due to AD (*n* = 799, CDR > 0.5) (McKhann et al., 2011). Table 1 shows the clinical and sociodemographic characteristics of the sample used for this study.

Model performance was evaluated across three simulated FL scenarios designed to assess the effects of data imbalance and heterogeneity (Table 3). In scenario 1, where institutions contributed equally and class distributions were identical, local models achieved comparable balanced accuracies (0.71 and 0.73). Under FL, performance improved modestly: balanced accuracy increased to 0.73 and 0.79, and sensitivity rose from 0.69 to 0.82 at Node 1 and from 0.71 to 0.83 at Node 2, reflecting better detection of CI cases. Precision remained consistently high (>0.95) across all models. Specificity was stable at Node 2 but declined slightly at Node 1 (0.73 to 0.64). Importantly, FL enhanced overall discriminative ability, with AUC rising from 0.76 to 0.81 at Node 1 and from 0.82 to 0.87 at Node 2, and F1-score increasing from 0.80 to 0.88 at Node 1 and from 0.82 to 0.90 at Node 2 (Table 4).

**Table 3:** Sample sizes and corresponding train-test splits for each node across three scenarios.

Scenario	Node	Total sample size	Train set	Test set
Scenario 1	Node 1	1,119	783	336
	Node 2	1,120	784	336
Scenario 2	Node 1	223	156	67
	Node 2	2,016	1,411	605
Scenario 3	Node 1	1,395	1,032	376
	Node 2	831	548	283

**Table 4:** Classification performance metrics for cognitive impairment prediction across three scenarios settings.

Scenario	Training	Balanced accuracy		Precision		Sensitivity		Specificity		F1-score		AUC	
		Node 1	Node 2	Node 1	Node 2	Node 1	Node 2	Node 1	Node 2	Node 1	Node 2	Node 1	Node 2
Scenario 1	Local	0.71	0.73	0.96	0.96	0.69	0.71	0.73	0.76	0.80	0.82	0.76	0.82
	FL	0.73	0.79	0.95	0.97	0.82	0.83	0.64	0.76	0.88	0.90	0.81	0.87
Scenario 2	Local	0.51	0.76	1.00	0.97	0.02	0.76	1.00	0.77	0.03	0.85	0.59	0.83
	FL	0.80	0.70	0.95	0.95	0.93	0.90	0.67	0.51	0.94	0.92	0.89	0.84
Scenario 3	Local	0.70	0.74	0.98	0.93	0.71	0.70	0.69	0.77	0.82	0.80	0.79	0.81
	FL	0.71	0.72	0.99	0.95	0.57	0.60	0.85	0.85	0.73	0.73	0.81	0.84

Results are reported for node 1, node 2, and the federated learning (FL) model.

In scenario 2, with a strong imbalance in dataset size, with Node 1 contributing only 10% of the total data (Table 3), the local model at that node struggled to identify CI cases, with a sensitivity of just 0.02 and a balanced accuracy of 0.51. This reflects the model’s inability to generalize with such limited training data. However, when applying FL, performance at Node 1 improved dramatically: balanced accuracy rose to 0.80 and sensitivity to 0.93, F1-score to 0.94, and AUC to 0.89, showing that FL training enabled robust CI detection. At Node 2, which had a much larger dataset, high performance was maintained under FL (balanced accuracy 0.70, F1-score 0.92, AUC 0.84), underscoring the scalability of the approach and its ability to distribute benefits fairly across nodes of unequal size. Notably, specificity decreased at both nodes (from 1.00 to 0.67 at Node 1 and from 0.77 to 0.51 at Node 2), reflecting a trade-off where improved CI detection came at the expense of more CU misclassifications.

In scenario 3, class distribution was imbalanced across institutions, with Node 1 having 97% CI cases and only 3% CU cases, while Node 2 had a more representative mix (Table 3). Local models performed similarly to scenario 1, with balanced accuracies of 0.70 (Node 1) and 0.74 (Node 2). Under FL, Node 1 maintained similar balanced accuracy (0.71) but with shifted trade-offs: sensitivity decreased from 0.71 to 0.57 and F1-score from 0.82 to 0.73, while specificity improved from 0.69 to 0.85, suggesting that FL training allowed the model to better identify CU cases despite their scarcity. Node 2 showed stable balanced accuracy (0.72) and slightly higher AUC (0.84), indicating that federated training conferred benefits, particularly in recognizing minority-class CU samples without loss of overall classification power.

The complete confusion matrices for each scenario, including local and FL models, are provided in the Supplementary Table S1.

## 4 Discussions and conclusions

In this study, an MLP model was trained using speech data and developed using a federated network to predict CI, specifically distinguishing between cognitively healthy individuals, and patients with MCI and dementia. FL was tested to confirm its ability to enable collaboration among multiple institutions while safeguarding patient privacy. This decentralized approach is particularly advantageous in healthcare, where privacy is a significant concern, as it facilitates multi-site model training without requiring the exchange of raw data (Zhang et al., 2024).

The choice of MLP reflects the trade-offs inherent in FL. While more complex architectures (e.g., CNNs, RNNs, Transformers) may achieve higher performance in centralized settings, they are computationally demanding and more prone to divergence under heterogeneous data. MLPs, in contrast, are lightweight, stable, and communication-efficient, aligning well with structured acoustic features such as the 176 descriptors used here. Empirically, this balance proved effective: the MLP achieved strong AUC and F1-scores across scenarios, while FL improved sensitivity in low-data nodes and enhanced generalization under class imbalance. Thus, the MLP offered the most pragmatic compromise between expressive power, robustness, and scalability (Zhang et al., 2024).

Equally important was the choice of aggregation strategy. The combination of FedAvg and IDA proved particularly suitable, as it consistently improved performance under challenging conditions. In Scenario 2, where the smaller institution held only 10% of the data, local training nearly failed (sensitivity = 0.02), yet the federated model raised sensitivity to 0.93 and AUC to 0.89, showing clear benefits for underrepresented nodes. In Scenario 3 with severe class imbalance, this combination also improved specificity (0.69 to 0.85) and AUC (0.79 to 0.81), enhancing recognition of CU cases. FedAvg offered scalability and efficiency, while IDA mitigated heterogeneity, together providing a balanced and pragmatic solution (Meerza et al., 2022). Other strategies, such as FedProx or Scaffold, address non-IID conditions by adding proximal terms or variance-reducing corrections, but they require additional hyperparameter tuning and may increase communication or computation costs, making them less practical for early-stage clinical deployment. Similarly, clustered FL can personalize models for subgroups, but at the expense of interpretability and cross-site comparability, which are essential in healthcare. Against this backdrop, FedAvg combined with IDA offered the best balance between simplicity, robustness, and feasibility (Qi et al., 2023).

Overall, the federated MLP consistently achieved AUC values above 80%, demonstrating reliable discrimination between CU and CI. Its greatest impact emerged under scarcity and imbalance: in Scenario 2, balanced accuracy rose from 0.51 to 0.80 and sensitivity from 0.02 to 0.93, while in Scenario 3, FL improved specificity and AUC, aiding recognition of minority CU cases. By contrast, gains in balanced settings (Scenario 1) were modest, indicating that FL’s strength lies less in boosting absolute accuracy than in ensuring fair and robust performance across heterogeneous institutions. These results highlight FL as a privacy-preserving framework that promotes equity, allowing smaller or skewed sites to contribute meaningfully without being disadvantaged.

Taken together, the findings confirm that the decentralized nature of FL provides a scalable and privacy-preserving solution for CI detection, enabling robust model development without direct data sharing (Meerza et al., 2022). By addressing dataset size inequality and class imbalance, FL supports fairer predictions across diverse populations and is particularly valuable for detecting subtle cognitive changes in early disease stages (Mateus et al., 2023). Beyond modest performance improvements, its benefits extend to equity across institutions, compliance with privacy regulations, enhanced security through decentralization, and scalability across heterogeneous clinical environments.

However, several limitations should be acknowledged. First, although the cohort included 2,239 participants, all data came from a single institution. The simulated disparities between two virtual sites with harmonized preprocessing cannot replicate true cross-site heterogeneity, where differences in devices, demographics, diagnostic protocols, and annotation quality are more pronounced. Real-world clinical settings also introduce variability in speech tasks, legal constraints, and annotation standards, which may affect both convergence and fairness. These factors highlight the need for validation beyond controlled simulations.

Second, while FL demonstrates strong potential for collaborative and privacy-preserving modeling, it is not immune to security vulnerabilities. Risks such as model inversion attacks, membership inference, and data leakage could compromise patient confidentiality or model integrity. Mitigating these risks will require safeguards including differential privacy, secure aggregation, and adversarial robustness strategies, alongside standardized monitoring and quality control, to ensure that FL systems remain safe and trustworthy in clinical deployment (Vasa et al., 2024).

Future work should prioritize multi-institutional pilots to evaluate scalability across larger networks (e.g., 5–10 sites) using diverse devices, protocols, and languages, thereby testing cross-linguistic generalizability. These studies should compare aggregation strategies (e.g., FedAvg vs. adaptive methods) and investigate personalization, domain adaptation, and harmonization frameworks to balance local adaptation with global performance. Evaluation must extend beyond AUC and F1-scores to include fairness, calibration, and node-specific robustness, ensuring that benefits are not driven solely by data-rich sites. Expanding FL to geographically distributed cohorts and incorporating multimodal data sources, such as cognitive assessments, neuroimaging, and electronic health records, will further enhance diagnostic accuracy and clinical applicability. To achieve this, key steps include: (1) establishing multi-institutional pilots under real-world conditions, (2) developing standardized protocols for data harmonization and evaluation across sites, (3) creating interoperable, privacy-compliant FL infrastructure aligned with regulations, and (4) building decision-support tools to integrate FL outputs into clinical workflows. Establishing harmonized speech protocols and secure, standardized infrastructure will be essential to move beyond experimental validation and enable safe deployment in healthcare systems (Dhade and Shirke, 2024).

Overall, this study highlights the promise of FL as a scalable, secure, and effective approach for early detection of cognitive impairment. By enabling privacy-preserving collaboration across institutions, FL addresses key challenges in digital health research and opens new avenues for developing accessible and equitable diagnostic tools for neurodegenerative diseases.

## Data Availability

The datasets presented in this article are not readily available because the datasets generated and/or analyzed contain human privacy-sensitive data but are available from the corresponding author on reasonable request. Requests to access the datasets should be directed to Sergi Valero, svalero@fundacioace.org.
