# Supplementary material for: Federated learning for cognitive impairment detection using speech data
Source: Front Artif Intell. 2025 Oct 9;8:1662859. doi: 10.3389/frai.2025.1662859 (PMC12548476; doi:10.3389/frai.2025.1662859)
Supplement: Supplementary file 1 [file Table_1.docx]

| Scenario | Train | Test | Confusion matric | | | |
| --- | --- | --- | --- | --- | --- | --- |
|  |  |  | True positive | False negative | False positive | True negative |
| 1 | Node 1 | Node 1 | 210 | 93 | 9 | 24 |
|  | Node 2 | Node 2 | 214 | 89 | 8 | 25 |
|  | FL | Node 1 | 248 | 55 | 12 | 21 |
|  |  | Node 2 | 252 | 51 | 8 | 25 |
| 2 | Node 1 | Node 1 | 1 | 57 | 0 | 9 |
|  | Node 2 | Node 2 | 415 | 133 | 13 | 44 |
|  | FL | Node 1 | 54 | 4 | 3 | 6 |
|  |  | Node 2 | 491 | 57 | 28 | 29 |
| 3 | Node 1 | Node 1 | 256 | 107 | 4 | 9 |
|  | Node 2 | Node 2 | 162 | 68 | 12 | 41 |
|  | FL | Node 1 | 208 | 155 | 2 | 11 |
|  |  | Node 2 | 138 | 92 | 8 | 45 |

**Table S1.** Confusion matrices for all three scenarios (training and test results per node and federated model). Each cell shows the number of true positives, false negatives, false positives, and true negatives.
